# Prevalence and determinants of heavy episodic drinking among adults in Kenya: analysis of the STEPwise survey, 2015

**DOI:** 10.1186/s12889-018-6057-6

**Published:** 2018-11-07

**Authors:** Anne Kendagor, Gladwell Gathecha, Melau W Ntakuka, Philip Nyakundi, Samuel Gathere, Dorcas Kiptui, Hussein Abubakar, Oren Ombiro, Pamela Juma, Christine Ngaruiya

**Affiliations:** 1 Division of Non-Communicable Diseases, Ministry of Health, Nairobi, Kenya; 2 Alcohol control focal point, Ministry of Health, Nairobi, Kenya; 3 Field Epidemiology and Laboratory Training Program, Ministry of Health, Nairobi, Kenya; 4 Improving Public Health Management for Action Program, Ministry of Health, Nairobi, Kenya; 5 Kenya Medical Research Institute (KEMRI), Nairobi, Kenya; 6 African Population and Health Research Center, Nairobi, Kenya; 7 Yale School of Medicine, New Haven, CT USA

**Keywords:** Alcohol, Episodic drinking, Consumption, Control

## Abstract

**Background:**

Globally, alcohol consumption contributes to 3.3 million deaths and 5.1% of Disability Adjusted Life Years (DALYs), and its use is linked with more than 200 disease and injury conditions. Our study assessed the frequency and patterns of Heavy Episodic Drinking (HED) in Kenya. HED is defined as consumption of 60 or more grams of pure alcohol (6+ standard drinks in most countries) on at least one single occasion per month. Understanding the burden and patterns of heavy episodic drinking will be helpful to inform strategies that would curb the problem in Kenya.

**Methods:**

Using the WHO STEPwise approach to surveillance (STEPS) tool, a nationally representative household survey of 4203 adults aged 18–69 years was conducted in Kenya between April and June 2015. We used logistic regression analysis to assess factors associated with HED among both current and former alcohol drinkers. We included the following socio-demographic variables: age, sex, and marital status, level of education, socio-economic status, residence, and tobacco as an interaction factor.

**Results:**

The prevalence of HED was 12.6%. Men were more likely to engage in HED than women (unadjusted OR 9.9 95%, CI 5.5–18.8). The highest proportion of HED was reported in the 18–29-year age group (35.5%). Those currently married/ cohabiting had the highest prevalence of HED (60%). Respondents who were separated had three times higher odds of HED compared to married counterparts (OR 2.7, 95% CI 1.3–5.7). Approximately 16.0% of respondents reported cessation of alcohol use due to health reasons. Nearly two thirds reported drinking home-brewed beers or wines. Tobacco consumption was associated with higher odds of HED (unadjusted OR 6.9, 95% CI 4.4–10.8); those that smoke (34.4%) were more likely to engage in HED compared to their non-smoking counterparts.

**Conclusion:**

Our findings highlight a significant prevalence of HED among alcohol drinkers in Kenya. Young males, those with less education, married people, and tobacco users were more likely to report heavy alcohol use, with male sex as the primary driving factor. These findings are novel to the country and region; they provide guidance to target alcohol control interventions for different groups in Kenya.

## Background

Globally, harmful alcohol consumption contributes to 3.3 million deaths and 5.1% of disability–adjusted life years (DALYs) [1]. Harmful alcohol use is associated with more than 200 diseases and injury conditions. Some of the diseases associated with harmful alcohol use include alcohol dependence, liver cirrhosis, cancers and injuries [1]. A study done on the contribution of the six preventable risk factors to achieving 25% reduction of non-communicable disease (NCD) mortality by 2025 (25 by 25) found that no WHO region will meet the target if the current rate of mortality continues to be reported [2]. Heavy Episodic Drinking affects 12 out of the 17 Sustainable Development Goals through its multiple public health, social and economic impacts [3]. The global strategy to reduce the harmful use of alcohol recognizes the close links between the harmful use of alcohol and socioeconomic development.

The negative health effects associated with alcohol use have been linked to Heavy Episodic Drinking (HED). The WHO defines binge drinking as consumption of 60 or more grams of pure alcohol (6+ standard drinks) on at least one single occasion at least once in a month [1]. HED has been linked to a myriad of both acute and more long-term negative health outcomes such as alcohol poisoning, injuries, pancreatitis, hypertension, ischemic heart disease, and cerebrovascular disease [4–6]. Worldwide, 7.5% of alcohol drinkers have Heavy Episodic Drinking occasions on a monthly basis [7]. In England, for example, it was estimated that 34% of men and 28% of women drink more than the recommended amount of alcohol at least 1 day of the week and 18% of men and 12% of women drink heavily [8]. In the United States, it is estimated that in 2015, 26.9% of people aged 18 and older reported to have engaged in Heavy Episodic Drinking [9].

In Kenya, about 35.7% of all alcohol users reported that they had diverted resources in order to buy alcohol [10]. It is therefore an obstacle to development [3]. There is a close a correlation between HED and infectious diseases such as HIV/AIDs. A study done in Mombasa, Kenya found that 33% of the respondents engaged in Heavy Episodic Drinking, and were more likely to report unprotected sex and sexual violence [11]. Furthermore, consumption beyond the recommended limits could result in alcohol dependency and other mental health or substance use disorders. In a study done to identify the development of alcohol disorders, it was found out that, of those who began drinking at ages 11–12 years, 13.5% met a criteria for diagnosis of alcohol abuse, and 15.9% had a diagnosis of dependence [12]. A NACADA survey indicated that 13% of Kenyans who drink alcohol have developed dependency [10].

It is critical to understand the patterns and risk factors associated with unhealthy consumption of alcohol in Kenya and similar settings given alcohol use disorders and associated conditions are on the rise. Our study is the first of its kind with a nationally representative sample done in Kenya to assess frequency and patterns of alcohol use, including addressing “unrecorded” alcohol consumption in the country. Understanding patterns of risky alcohol consumption will be helpful to inform strategies that would curb the problem in Kenya.

Understanding patterns of HED will be helpful to inform public health strategies of alcohol being a risk factor for many diseases and conditions. The study emphasizes on HED whose health and social consequences that have been shown to be more detrimental than regular alcohol consumption [13]. Findings from the study are therefore important to enhance alcohol control prioritization among this specific risk group. It is imperative that public health resources be channeled to targets groups at risk of indulging in HED [14].

## Methods

This paper is based on cross-sectional data from the WHO STEPwise approach to surveillance (WHO STEPS) study that was conducted in Kenya between April and June 2015 [15]. Participants were identified using a three-stage cluster sampling approach involving selection of clusters, households and individuals from the National Bureau of Statistics household-based sampling frame (NASSEP V) [16]. Further details on the sampling procedures are described in the Kenya WHO STEPS report [15]. The individual identified as the head of household at the time of contact responded to the survey. Written informed consent was obtained from the selected individual. Individuals were eligible to participate if they were aged 18–69 years old. Those that refused consent were excluded from the study. Data were collected using the STEPS instrument, a cross-culturally validated survey tool used to assess burden of lead non-communicable diseases and associated lifestyle risk factors.

### Independent variables

The socio-demographic factors age, sex, number of years of education, occupation, wealth index, residence, and tobacco use were considered as independent variables. The variable wealth index is used as a marker of overall socioeconomic status, and was created as part of the original STEPS study [15]. It is presented in five quintiles. The wealth index was generated using the multivariate statistical technique (Principal Components Analysis), according to methodology outlined by the DHS (Demographic and Health Surveys) program [16]. Principal components are weighted averages of the variables used to construct them. Among all weighted averages, the first principal component is usually the one that has the greatest ability to predict the individual variables that make it up, where prediction is measured by the variance of the index. Variables included in wealth index determination were: type of dwelling, ownership of the dwelling, construction materials of the dwelling, source of cooking fuel, source of lighting fuel, household possessions/ goods, source of water for household consumption, and type of sanitation facility, as indicated by the Kenya STEPS report [15].

### Dependent variables

The primary dependent variable was Heavy Episodic Drinking (HED). The variable was created from the question that queried the number of drinks consumed by respondents in the past 30 days. Those reporting six drinks or more were categorized as having engaged in HED, per standard WHO definition previously discussed [6]. We also assessed alcohol consumption within the past 30 days and the past 12 months, as well as the average number of standard alcohol drinks consumed per sitting, and average number of binge days. The average number of binge days was determined from responses on a question that asked how many times six or more standard drinks were consumed in a single occasion over the past 30 days. We additionally inquired about having stopped drinking for more than 12 months due to health reasons and this was determined by the question “Have you ever stopped drinking due to health reasons, such as a negative impact in your health or on the advice of your doctor or other health worker”.

### Descriptive analysis

For descriptive analysis, frequencies and proportion are presented; the percentages presented were weighted for the population in line with the weighting factors used for the survey as discussed further in the STEPS survey report [15].The distribution of tobacco use amongst alcohol users, given established association [17] was also assessed in bivariate analysis.

### Multivariable analysis

While we hypothesized that sociodemographic variables would have an association with Heavy Episodic Drinking, this is based on literature outside of our setting, and the discovery of novel relationships in our population was also of interest. Given this, all sociodemographic variables were included in final regression models using a stepwise process with inclusion in the final model if the independent variable was found to have a statistically significant relationship with HED. Based on existing literature on the topic, we hypothesized that those that are younger, men, those with fewer number of years of education, lower wealth index, and those living in the urban areas were more likely to be involved in heavy episodic drinking. Our alternative hypothesis then was that social demographic variables had influence on heavy episodic drinking. A multivariable regression model was done to test these relationships.

Occupation was not included in the logistic regression analysis because the original coding of the variable (categories chosen for the survey) were not felt to be good differentiators among the respective categories, and thus not indicative of socioeconomic status. The variable ‘tobacco use’ was hypothesized to be an interaction factor given the known association with aforementioned socio-demographic factors and alcohol use. It was therefore felt to be along the causal pathway and treated as such. Testing for interaction between each of the independent variables in the model, tobacco use, and the outcome HED, was done. Statistical evidence for interaction was found to be present between sex, tobacco use, and HED, so results are presented showing only the effects of the interaction on HED (and not individually for the effects of sex on HED, or the effects of tobacco use on HED). Tables of finding present unadjusted and adjusted odds ratios for variables included in the final model. For those variables that were not included in the final model, only the unadjusted odds ratios are presented. Finally, both unadjusted and adjusted odds ratios are presented with associated 95% confidence intervals (CIs).

## Results

A total of 4203 respondents were included in this analysis. In our findings, men comprised the majority of respondents (60%). Nearly 40% of respondents reported having ever consumed alcohol before (Table 1). Heavy Episodic Drinking was reported by 12.7% of the respondents. Two out of five (40.4%) respondents reported to have consumed alcohol within the past 7 days. The highest mean consumption of alcohol was recorded on Saturday (5.3) drinks. The majority of unrecorded alcohol consumed was home-brewed beer wine or spirits, as shown in. Sixteen percent of respondents who had not drunk in the preceding 12 months reported having stopped drinking secondary to health reasons.

**Table 1:** Characteristics and patterns of alcohol use in Kenya

	Male	Female	Total Number (n^b^, %^a^)
Ever consumed alcohol/Ever user			
Yes	936 (58.5)	456 (19.7)	1392 (38.6)
No	738 (41.5)	2073 (80.3)	2811 (61.4)
Heavy Episodic Drinking			
Yes	325 (20.6)	59 (2.5)	384 (12.6)
No	1349 (79.4)	2471 (97.5)	3820 (88.6)
Period of alcohol consumption among ever consumers			
Within past 7 days	451 (27.3)	105 (4.6)	556 (40.4)
Within past 30 days	536 (81.9)	129 (53.7)	665 (47.8)
Within past 12 months	667 (69.9)	210 (51.4)	877 (63.0)
Mean consumption of one or more standard drink among current drinkers (95% CI) ^a^			
Monday	1.1 (0.8, 1.3)	0.7 (0.3, 1.1)	1.0 (0.7, 1.3)
Tuesday	0.8 (0.6, 1.0)	0.6 (0.3, 0.9)	0.7 (0.6, 0.9)
Wednesday	1.0 (0.8, 1.3)	0.8 (0.2, 1.4)	1.0 (0.7, 1.2)
Thursday	1.0 (0.7, 1.2)	0.6 (0.2, 1.0)	0.9 (0.7, 1.2)
Friday	4.7 (3.4, 5.9)	2.8 (0.0, 5.6)	4.4 (3.1, 5.7)
Saturday	5.8 (4.5, 7.1)	2.4 (0.4, 4.4)	5.3 (4.0, 6.5)
Sunday	1.2 (1.0, 1.4)	1.1 (0.7, 1.6)	1.2 (1.0, 1.4)
Types of unrecorded alcohol consumed			
Home-brewed spirits	110 (54.8)	20 (59.1)	130 (55.4)
Home-brewed beer or wine	106 (60.1)	31 (80.9)	137 (62.8)
Alcohol not intended for drinking	3 (1.9)	0	3 (100)
Other untaxed alcohol	2 (0.2)	1 (2.2)	3 (0.5)
(Self) imported alcohol	0	1 (4.1)	1 (0.5)
Former drinkers stopped drinking due to health reasons			
Yes	53 (22.2)	18 (5.3)	71 (16.0)
No	217 (77.8)	229 (94.7)	446 (84.0)
Total	1674 (39.8)	2529 (60.2)	4204

^a^Weighted % or mean representing population level

^b^Except for first row, n is total number of participants who had ever consumed alcohol, which is 1392. However, the total may be less for individual variables, due to missing data for some questions

Alcoholic drink that is homebrewed alcohol (excluding changaa, busaa or muratina) or any alcohol not intended for drinking in the past 12 months

Among those who reported engaging in HED, the majority were men (88.5%) as shown in Table 2. HED was highest among those age 18–29 years (35.2%). Nearly half of respondents (46.9%) engaging in HED reported having only a primary education or less. Married respondents were most likely to report recent drinking and HED, whereas those that were single reported a higher number of average drinks per sitting. Respondents who were non-government employees and self-employed demonstrated the highest prevalence of HED, 37.7% and 19.3%, respectively. Those in the rural areas had a slightly higher prevalence of drinking as compared to urban counterparts. There were fewer smokers than non-smokers engaged in HED (34.4%), however as compared to their non-smoking counterparts; they had higher odds of engaging in HED (unadjusted OR 6.9, 95% CI 4.4–10.8).

**Table 2:** Breakdown of heavy alcohol use by sociodemographic characteristics in Kenya

Characteristics	Consumed alcohol in the past 30 days (n, %) *N* = 665	Consumed alcohol in the past 12 months (n, %) *N* = 877	Average number of drinks per sitting (mean, 95% CI) *N* = 662	Average number of “binge” days (mean, 95% CI) *N* = 646	Presence of “heavy episodic drinking” (n, %) *N* = 384
Age					
18–29	156 (35.4)	240 (40.7)	9 (7,11)	3 (2,4)	83 (35.2)
30–39	215 (28.4)	283 (261)	11 (9,13)	5 (4,7)	125 (28.6)
40–49	138 (19.6)	166 (18.0)	9 (7,11)	5 (3,8)	90 (21.0)
50–59	88 (10.3)	103 (9.4)	8 (6,10)	4 (2,6)	46 (8.7)
60–69	68 (6.3)	85 (5.8)	13 (5,20)	4 (2,7)	40 (6.4)
Sex					
Men	536 (85.4)	667 (79.3)	10 (9,11)	5 (4,6)	325 (88.5)
Women	129 (14.6)	210 (20.7)	8 (5,11)	2 (1,2)	59 (11.5)
Education level					
No Education	73 (8.5)	92 (8.3)	11 (5,17)	3 (2,4)	44 (8.3)
Primary	300 (43.3)	390 (42.6)	9 (7,12)	4 (3,5)	166 (38.2)
Secondary	172 (28.5)	221 (28.3)	10 (8,12)	5 (4,7)	106 (32.9)
Tertiary	120 (19.7)	174 (20.8)	9 (7,11)	3 (2,5)	68 (20.6)
Marital status					
Currently married/Cohabiting	433 (63.8)	554 (61.1)	9 (8,11)	5 (4,6)	242 (60.4)
Never married	118 (21.2)	175 (25.0)	11 (10,13)	3 (2,5)	75 (25.0)
Formerly married/widowed	114 (15.0)	148 (14.0)	10 (7,13)	4 (3,5)	67 (14.6)
Occupation					
Government employee	76 (13.2)	94 (12.2)	9 (7, 11)	5 (3,7)	47 (14.5)
Non-government employee	106 (19.0)	144 (18.6)	10 (8, 12)	4 (2,5)	67 (19.3)
Self-employed	311 (43.0)	388 (39.9)	8 (7, 9)	4 (3,6)	160 (37.7)
Non-paid/volunteer	2 (0.3)	5 (0.5)	7 (7, 8)	0 (0,1)	2 (0.5)
Student	21 (5.5)	34 (7.0)	8 (5, 11)	2 (1,3)	10 (5.9)
Homemaker	54 (6.0)	88 (9.5)	10 (5, 14)	2 (1,3)	31 (5.8)
Retired	17 (1.9)	18 (1.5)	20 (8, 32)	8 (1,15)	15 (2.8)
Unemployed able to work	75 (10.6)	97 (10.0)	16 (11, 20)	6 (3, 9)	50 (13.0)
Unemployed unable to work	3 (0.4)	9 (0.8)	9 (4, 14)	0 (0, 1)	2 (0.5)
Wealth quintile					
1 Poorest	127 (17.4)	157 (16.5)	13 (8, 18)	3 (2,5)	66 (15.3)
2 Second	137 (18.5)	176 (18.7)	10 (8,12)	6 (4,9)	73 (16.8)
3 Middle	122 (17.0)	153 (16.5)	8 (7,10)	3 (2,4)	73 (15.8)
4 Fourth	123 (18.9)	170 (17.8)	8 (6,10)	4 (2, 6)	76 (18.2)
5 Richest	156 (28.2)	221 (30.5)	10 (9,11)	4 (2,6)	96 (33.8)
Residence					
Rural	311 (54.3)	405 (53.2)	10 (8,12)	4 (3,5)	179 (52.0)
Urban	354 (45.7)	472 (46.8)	9 (8,10)	4 (3,6)	205 (48.0)
Currently smoking					
Yes	217 (30.7)	251 (28.0)	12 (10,14)	7 (5,9)	150 (34.4)
No	448 (69.3)	624 (71.9)	9 (7,10)	3 (2,4)	234 (65.6)

*Weighted % or mean representing population level

Table 3 shows the covariates associated with HED identified using logistic regression, as described in the methods section. When assessing the effects of sociodemographic status on HED, we found that all of our hypothesized variables: age, sex, number of years of education, residence, and current smoking were found to have statistically significant relationships with HED. Adults aged 40–49 years old were nearly twice as likely to be engaged in HED as compared to their younger counterparts in the 18–29-year age group (OR 1.9, 95% CI 1.0–3.5). Men had nearly ten times higher odds of engaging HED as compared to women. Finally, there was evidence of interaction between sex and current smokers on odds of HED, and non-smokers had around eight time’s higher odds of HED as compared to smokers (OR 7.9, 95% CI 4.1–15.5). The effects of number of years of education, wealth quintile, and residence were not found to be statistically significant after controlling for confounding. Unrecorded alcohol users made up 19.6% (*N* = 274/1392) of all alcohol consumers, and the majority of those reporting HED.

**Table 3:** Covariates associated with “heavy episodic drinking” in Kenya

	Unadjusted Odds Ratio (95% CI)	*P*-value	Adjusted Odds Ratio^a^ (95% CI)	P-value
Age (per 10 years)	1.15 (1.03,1.29)	0.01	1.15 (0.98,1.34)	0.08
Age categories		0.10		
18–29	1.0			
30–39	1.7 (1.1,2.7)	0.02		
40–49	1.9 (1.0,3.5)	0.05		
50–59	1.2 (0.8,1.8)	0.46		
60–69	1.7 (1.0,3.0)	0.07		
Sex				
Men	9.9 (5.3,18.8)	<.0001		
Women	1.0			
Sex^a^currently smoking				0.006
Smoker subgroup: man vs. woman			2.0 (0.7,5.3)	0.19
Non-smoker: man vs. woman			7.9 (4.1,15.5)	< 0.0001
Marital status		0.31		0.26
Currently married/ Cohabiting	1.0		1.0	
Never married	1.2 (0.8,1.8)	0.44	0.9 (0.6,1.4)	0.66
Formerly married/widowed	1.4 (0.8,2.5)	0.19	1.8 (0.9,3.5)	0.10
Education level		0.12		0.50
No education	1.0	–	1.0	–
Primary	1.6 (0.9,2.9)	0.11	1.2 (0.6,2.3)	0.57
Secondary	2.0 (1.04,3.9)	0.04	1.5 (0.8,2.8)	0.21
Tertiary	2.5 (1.1,5.6)	0.02	1.6 (0.7,3.8)	0.28
Wealth quintile		0.02		0.02
Poorest	1.00		1.0	
Second	1.0 (0.5,1.9)	0.92	0.8 (0.4,1.6)	0.45
Middle	1.0 (0.5,2.0)	0.90	0.7 (0.4,1.5)	0.38
Fourth	1.2 (0.6,2.4)	0.62	0.8 (0.4,1.8)	0.64
Richest	1.9 (0.9,4.1)	0.07	1.7 (0.8,3.8)	0.18
Residence				
Rural	0.6 (0.4,1.0)	0.04	1.0 (0.7,1.5)	0.86
Urban	1.00			
Currently smoking				
Yes	6.9 (4.4, 10.8)	<.0001		
No	1.00			

^a^The final model (adjusted model) included age, marital status, education, wealth quintile, residence, gender, currently smoking, and interaction of gender and currently smoking. The interaction of each predictor with smoking status for HED outcome was tested, however only interaction of gender by smoking remained significant. Because interaction term of gender by smoking is significant, the main effects of smoking and gender are not presented. Instead, gender effect is presented stratified by smoking status (the interaction indicates that the effect of gender differs significantly by smoking status)

## Discussion

This study is the first nationally representative study to our knowledge that examines the socio-demographic and behavioral determinants of Heavy Episodic Drinking among adults in Kenya. We found 12.7% of Kenyans to be involved in HED, with the majority (63.0%) being current drinkers or having reported consumption within the past 12 months. This is lower than what has been reported in other East African countries. Uganda reported a prevalence of HED of 16.7% [18], and Rwanda has a prevalence of 30.5% among males and 17.1% among females [19]. The lower rates could be due to a higher cost of living found in Kenya which includes the price of commodities such as alcohol, making it less accessible [20]. Because of these findings, it is prudent to put in measures such as intersectoral polices, community engagement and reorientation of health services to ensure that there is no increase in prevalence [21].

Our findings showed that younger populations were most likely to engage in HED. This is comparable to findings of other studies that have shown a higher prevalence among university age students, and the development of alcohol use disorders implied by starting this young [4, 22, 23]. With more than one third of Heavy Episodic Drinking occurring among Kenyan youth, the importance of targeting interventions towards them cannot be overstated. The addictive potential of alcohol is high [12] and cessation is challenging. Therefore, it is key that emphasis is placed on preventing onset of alcohol use particularly at this vulnerable age. Such measures include prohibition of advertising, which are included in the Alcoholics Drinks Control Act [24]. The provisions of this law are, however, weak and amendments are required to make it more comprehensive.

Men have higher odds of engaging in HED compared to women. This could be attributed to societal and cultural issues where alcohol is rewarding for men, whereas it is shameful for women. Additionally, men in Kenya are more likely to be the breadwinner and have financial resources to afford alcohol. In the Kenya STEPs study sample: 18% of women reported having no formal schooling in comparison to just 7% of their male counterparts [15]. This is in line with other studies that have shown higher prevalence of HED among men in Uganda, Rwanda and Ethiopia [18, 19, 25].

In our study, the unadjusted odds of HED were higher in smokers compared to non-smokers. While there was a higher prevalence of non-smokers among those engaged in HED overall, there was still statistically significant evidence of smoking as a risk factor among those engaged in HED (those engaged in HED had higher odds of being a smoker than not), and a strong relationship at that. This demonstrates the importance of this risk factor among HED users. Previous studies have shown cigarette smoking to be the gateway to other drugs including alcohol. In a study done to assess the relationship between smoking and alcohol misuse [2], smoking appeared to increase the risk for alcohol misuse, including the likelihood of HED drinking, the amount consumed per episode and length of a drinking episode. This effect was however more prominent in non-daily smokers compared to daily smokers [17]. Another study by the same author among young adults found that intermittent smoking is more likely to occur during binge drinking [26]. Further studies are needed to better understand these findings. Nevertheless, public health policy makers should explore the possibility of instituting integrated interventions to reduce HED and smoking cessation as they have been shown to be effective [21, 27]. Additionally, the interaction of gender with smoking status for HED outcome was significant. Gender effect is presented stratified by smoking status: the interaction indicates that gender affects smoking status, and that gender explains more of the effect on likelihood of HED over smoking though both are significant.

This study shows that most Kenyans consume alcohol during the weekends. This could be attributed to a culture that supports social activities associated with alcohol and peer pressure [28]. This could also be due to the increased drinking hours allowed by the Alcoholics Drinks Control Act [24]. Policy-makers should be aware of a predominance of weekend drinking, especially occurring on Saturday, as deterrent policies are implemented such as traffic stops and fines which have been implemented in recent years to avert drunk driving ((SAMHSA), 2015).

Unrecorded alcohol is not subjected to any form of controls and regulations therefore it may have extremely high level of alcohol content above the recommended standard of an alcoholic drink. This can contribute immensely to alcohol harm and abuse by the user [29]. There is also the added risk of contamination with other poisons that can in turn lead to further ill health and death [30]. There is need to intensify public health education on the ill effects of unrecorded alcohol and increase enforcement against the selling and distribution of the same as stipulated in the Alcoholics Drinks Control Act [24].

Finally, a small proportion of current alcohol drinkers had stopped alcohol consumption due to health reasons (16%). It is therefore crucial to explore strategies for integration of alcohol control into health care [6, 31]. The National Strategy for prevention and control of Non Communicable Diseases has proposed measures to reduce the harmful use of alcohol but implementation of the strategy has been challenging due to financial constraints [32]. The country also stands to benefit from domesticating the Global Strategy to reduce the Harmful use of Alcohol, which is more comprehensive [6].

## Limitations

Study findings had several limitations. First, under reporting of alcohol intake, which was likely due to lack of social desirability of drinking behavior. Second, not being able to consider other factors associated with HED for example segregation of data by region, liquor outlet density, enforcement of law, attitudes, among others [10]. The major strength of this study was the national representation of the STEPs survey, including the wide geographic and population scope.

## Conclusions

Our findings highlight a significant prevalence of HED in Kenya. Alcohol use, particularly Heavy Episodic Drinking is prevalent in Kenya and is likely influenced by known socio-demographic factors that are amenable to evidence-based interventions. The laws and policies in place to control alcohol consumption should be appropriately implemented and enforced, while enhancing efforts to create awareness on the risks associated with harmful use of alcohol, particularly HED. There is need for strategic interventions among key populations in the society, which particularly include men, young adults, and tobacco users. Unique policies addressing unrecorded alcohol are needed in the country. Finally, the role of the health care setting in providing cessation strategies should be explored.

## Abbreviations

DHS: Demographic and Health Survey
HED: Heavy Episodic Drinking
MOH: Ministry of Health
NACADA: National Authority for the Campaign Against Alcohol and Drug Abuse
NCD: Non-Communicable diseases
WHO: World Health Organization
